# Leader Inclusiveness and Taking Charge: The Role of Thriving at Work and Regulatory Focus

**DOI:** 10.3389/fpsyg.2019.02393

**Published:** 2019-10-24

**Authors:** Nan Li, Qiu-Yun Guo, Hua Wan

**Affiliations:** ^1^School of Economics and Management, Nanjing University of Aeronautics and Astronautics, Nanjing, China; ^2^Department of Economic and Management, Taiyuan Institute of Technology, Taiyuan, China; ^3^School of Business Administration, Zhongnan University of Economics and Law, Wuhan, China

**Keywords:** leader inclusiveness, taking charge, thriving, regulatory focus, self-determination theory

## Abstract

Based on self-determination theory, this study developed and tested a moderated mediation model to explore the effect of leader inclusiveness on employee taking charge behavior in addition to the mediating role of thriving at work and the moderating role of regulatory focus in this relationship. We tested the model with a sample of 206 employees from a large state-owned firm in China. Structural equation modeling revealed that leader inclusiveness was positively related to thriving at work, which in turn influenced taking charge. Promotion focus significantly moderated the relationship between leader inclusiveness and thriving at work and the mediating effect of thriving at work. As a result, the relationship and its mediating mechanism became stronger when the promotion focus of employees was high.

## Introduction

Dynamic contextual changes and increased competitiveness generate greater demand for higher organizational performance, which depends on employee proactive behavior (Crant, 2000; Griffin et al., 2007; Grant and Ashford, 2008). In recent years, the concept of taking charge as a type of proactive behavior has received noticeable attention in theoretical research and practice (Burnett et al., 2013; Li et al., 2013; Li R. et al., 2016). This concept can be defined as “voluntary and constructive efforts, by individual employees, to effect organizationally functional change with respect to how work is executed within the contexts of their job, work units, or organizations” (Morrison and Phelps, 1999, p. 403). Although taking charge is certainly desirable, as it promotes organizational innovation, improves employee performance, and enhances organizational adaptability (Crant, 2000; Parker, 2000; Fuller et al., 2012), its risks of challenging the *status quo* and the delay in obtaining benefits (Morrison and Phelps, 1999; Fuller et al., 2012) discourage employees from adopting this behavior. Therefore, it is not only of theoretical significance to identify the effect of employees’ psychological impetus on taking charge but also of practical benefit to explore what leaders can do to stimulate this behavior.

Leadership has been considered a critical factor influencing employee taking charge behavior (Wu and Parker, 2017). For example, transformational leadership (Li et al., 2013, 2017; Parker and Wu, 2014), self-sacrificial leadership (Li R. et al., 2016), and empowering leadership (Li M. et al., 2016) have been examined in relation to taking charge. However, leader inclusiveness has not been highlighted in research on taking charge. Leader inclusiveness, which is at the core of relational leadership, focuses on leaders’ availability, openness, and accessibility in their interactions with employees (Carmeli et al., 2010), and it enables the effective functioning of diverse organizations often overlooked in other forms of leadership (Randel et al., 2017). With the growing trend toward employee proactive behavior to cope with the dynamic environment, understanding the relationship between leader inclusiveness and employee taking charge behavior is critical. This study aims to link leader inclusiveness and taking charge, taking into account several intervening variables.

**FIGURE 1:**
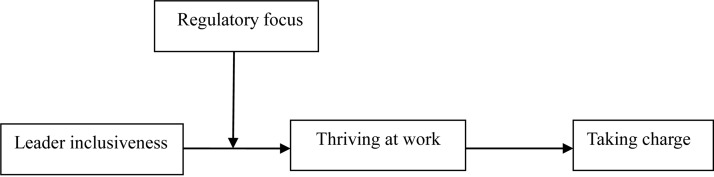
Research model.

Based on self-determination theory (SDT; Deci and Ryan, 1985, 2000), this study examines the mediating relationship between leader inclusiveness and taking charge via thriving at work, with a focus on individuals’ mental contingency of regulatory focus. By meeting employees’ basic human needs of belonging and being valued for their uniqueness (Randel et al., 2017), inclusive leaders ignite their passion for work and their intrinsic motivation to thrive (i.e., a psychological state in which employees experience both a sense of vitality and learning at work; Spreitzer et al., 2005). In turn, with a sense of thriving, individuals are likely to display agentic behaviors (e.g., taking charge) that continue or increase this state (Spreitzer et al., 2005). Hence, we suggest that thriving at work transmits the influence of leader inclusiveness to taking charge. In addition, we expect regulatory focus, which is defined as a chronic, individual disposition (Wallace et al., 2016), to affect reaction to leader inclusiveness. For example, individuals with high promotion focus (i.e., a type of regulatory focus that pays attention to nurturance needs, hopes, and aspirations) tend to be more affected by leader inclusiveness. Therefore, we also consider the moderating role of regulatory focus in this study. Overall, this study develops and examines a moderated mediation model to explore the relationship between leader inclusiveness and taking charge. Specifically, we hypothesize that thriving at work mediates the relationship between leader inclusiveness and taking charge, while regulatory focus moderates the relationship between leader inclusiveness and thriving at work (first stage moderation; Figure 1).

This study contributes to the literature in the following ways. First, we investigate the effect of leader inclusiveness on taking charge. Although previous research has discussed the effect of different leadership styles on taking charge (Parker and Wu, 2014; Lee, 2016; Li R. et al., 2016), little attention has been given to leader inclusiveness, which is a particular leadership style aimed at fostering an inclusive climate in the workplace. This study complements previous research on taking charge and extends the scope of the results of leader inclusiveness. Second, by examining the mediating effect of thriving at work, we explain the underlying mechanism of leader inclusiveness in taking charge. Based on the perspective of SDT, we explain the “black box” of how leader inclusiveness affects employee taking charge behavior. It suggests that leader inclusiveness enhances employee taking charge behavior because employees are able to experience a critical psychological state, such as thriving at work, which promotes their proactive behavior. Third, by exploring the moderating effect of regular focus based on the regulatory focus theory, our study identifies the unique boundary conditions for leader inclusiveness to influence thriving at work in general and how thriving at work affects the relationship between leader inclusiveness and taking charge.

## Theory and Hypotheses

### Leader Inclusiveness and Taking Charge

Inclusion has been studied in the field of education and social practice (Ryan, 1998, 2002, 2007). Ryan’s study was one of the few to investigate the effects of leader inclusiveness in an organizational context. Nembhard and Edmondson (2006) conceptualized leader inclusiveness as leaders’ words or actions that “indicate an invitation and appreciation for others’ contributions” (p. 947). In addition, Hantula (2009) proposed that inclusive leaders are “doing things with people, rather than to people” (p. 9). Furthermore, based on the definition of Nembhard and Edmondson (2006), Carmeli et al. (2010) suggested that inclusive leaders demonstrate availability and accessibility in their interactions with employees. Recently, Randel et al. (2017) proposed that leader inclusiveness refers to a set of behaviors facilitating employees’ feelings of being part of the organization while maintaining their sense of individuality. Scholars have explored the outcomes of leader inclusiveness. For example, leader inclusiveness can enhance employees’ innovation behaviors (Javed et al., 2017) and creativity (Carmeli et al., 2010), increase employees’ psychological safety (Nembhard and Edmondson, 2006; Javed et al., 2017), and promote work engagement (Choi et al., 2015). Following inclusive leaders, employees are included in discussions and decision-making, and their different perspectives and ideas can be adopted and implemented in the work process (Carmeli et al., 2010; Hirak et al., 2012). These positive outcomes and behaviors imply that leader inclusiveness can motivate employees to display proactive behavior and encourage employees to “immerse” themselves in their work.

The benefits of leader inclusiveness in taking charge are suggested by the tenets of SDT, which is a motivational framework that can be used to explain the motivation of individuals to adopt proactive behavior (Deci and Ryan, 1985, 2000). Gagné and Deci (2005) proposed that the degree of self-motivation of individuals depends on the extent to which their managers meet employees’ basic human needs by creating interpersonal relationships and providing support. By fostering an inclusive climate in the workplace, inclusive leaders give employees the freedom to work autonomously (Carmeli et al., 2010). In addition, inclusive leaders can help employees set up organizational identification, link self-development goals with work goals, and internalize the value and importance of taking charge (Gagné and Deci, 2005; Parker et al., 2010). Furthermore, inclusive leadership provides individuals with psychological nutriments to meet their basic human needs for autonomy (Deci and Ryan, 2000) and encourages employees to proactively participate in change-oriented behaviors. In particular, leader inclusiveness may have a salient influence on employee taking charge behavior in Chinese organizations, where employees usually maintain a certain psychological distance with their leaders (Chen et al., 2002; Cheng et al., 2003), do their in-role work to avoid making mistakes, and are not willing to adopt proactive behavior. Inclusive leaders who break rigid hierarchies between leaders and employees offer employees the opportunity to engage in decision-making and a way to grow. With emotional and practical support, employees are free and likely to take charge in their work. Therefore, we hypothesize the following:

H1. Leader inclusiveness is positively related to employee taking charge behavior.

### The Mediating Role of Thriving at Work

Thriving at work is defined as the mental state of joint experiences of vitality and learning at work (Spreitzer et al., 2005). Learning, which refers to individuals’ sense of acquisition and application of new knowledge and skills (Elliott and Dweck, 1988), and vitality, which is characterized by feelings of energy and aliveness when doing one’s work (Nix et al., 1999), represent the cognitive and affective components of thriving at work. Together, these two dimensions reflect individuals’ self-regulation by providing internal cues that help employees gauge whether they are developing in a positive direction. Moreover, as an adaptive function, thriving at work helps individuals adjust their work context to promote their development and growth (Spreitzer et al., 2005).

Based on SDT, we expect that inclusive leaders who do their best to create a positive work context to meet their employees’ basic psychological needs can help them thrive at work. First, inclusive leaders share information with their employees and encourage them to make decisions (Nishii and Mayer, 2009; Javed et al., 2017), increasing mutual trust between leader and employees and motivating employees to be more active and purposeful at work. Second, by providing their employees with work resources (i.e., information, knowledge, or affective support), inclusive leaders help them reduce barriers and stress at work and solve difficult problems encountered while working. Third, inclusive leaders tend to share their vision and identification with their employees. This increases employees’ sense of belonging and affective commitment to their leader and organization (Randel et al., 2017). In turn, this enhances employees’ autonomous motivation to thrive at work.

Thriving at work alone has significant implications for employee taking charge behavior. First, thriving employees are stimulated in their work (Spreitzer et al., 2005) and are more likely to engage in proactive actions to solve organizational problems and improve the *status quo* (Porath et al., 2011). Second, when employees are eager to learn and develop at work, they tend to acquire knowledge and information (Carver, 1998), through which they can get a more integrated picture of their work and become willing to change the current situation. Third, in this state, employees experience a positive mood and emotions, which facilitate their cognitive thinking and problem-solving abilities (Hirt et al., 1997; Prem et al., 2017). In addition, the experience of positive emotions provides psychological and social resources (Fredrickson, 2001) and encourages individuals to be more active and take charge. Based on the above discussion, leader inclusiveness helps employees thrive at work, which in turn promotes taking charge. Hence, thriving mediates the effect of leader inclusiveness on taking charge. In other words, leader inclusiveness can motivate employees to take charge, at least in part, because of a positive psychological state (e.g., thriving at work) stimulated by leader inclusiveness. Therefore, we hypothesize the following:

H2. Thriving at work mediates the relationship between leader inclusiveness and employee taking charge behavior.

### The Moderating Role of Regulatory Focus

Self-regulation refers to the process by which people seek to align with appropriate goals or standards (Brockner and Higgins, 2001). Higgins (1997, 1998) highlighted two orthogonal self-regulatory mindsets: promotion focus and prevention focus. In this study, we focused on the moderating effect of promotion focus associated with growth and developmental needs (Higgins, 1997, 1998). Individuals operating primarily with promotion focus are concerned about accomplishments, hopes, and aspirations and pursue an “ideal self” (Kark and Van Dijk, 2007). They are likely to exhibit “exploratory” behaviors (Förster et al., 2004) and to be more open to creativity and innovation (Cowden and Bendickson, 2018). Neubert et al. (2008) found a positive relationship between promotion focus and helping and creative behaviors, while Lanaj et al. (2012) proposed that promotion focus was associated with innovation.

In this study, promotion focus is expected to play a moderating role between leader inclusiveness and thriving at work. As inclusive leaders are particularly receptive to their employees’ suggestions and input and provide a suitable setting and climate in the workplace, employees with high promotion focus are more likely to respond and promote the positive effect of inclusive leaders on thriving at work. By following an inclusive leader, promotion-focused individuals show their enthusiasm for work and focus on promoting their expected outcomes (Neubert et al., 2008). They are more likely to regulate their psychological state and behaviors with the need for growth and development under an inclusive leader, helping them maintain positive feelings and a positive state of mind. In addition, as inclusive leaders tend to exhibit availability and accessibility to stimulate their employees’ self-development skills (Carmeli et al., 2010), promotion-focused individuals striving for aspirations and ideals are more likely to learn and work hard. In contrast, inclusive leaders have difficulty stimulating employees with low promotion focus to increase their positive self-development skills and motivate them to pursue ideals and aspirations. Thus, for employees with low promotion focus, the relationship between leader inclusiveness and thriving at work is weaker. Therefore, we hypothesize the following:

H3. Employees’ regulatory focus moderates the relationship between leader inclusiveness and thriving at work. Specifically, when promotion focus is high, the positive relationship between the two is enhanced.

By combining Hypotheses 1, 2, and 3, we further propose that employees’ promotion focus can moderate the indirect effect of leader inclusiveness on taking charge via thriving at work, thereby resulting in moderated mediation. For employees with high promotion focus, the effect of leader inclusiveness on thriving at work is stronger; therefore, the mediating effect of thriving at work between leader inclusiveness and taking charge becomes more positive.

H4. Employees’ regulatory focus moderates the mediated relationship between leader inclusiveness and taking charge via thriving at work such that this mediated relationship will be stronger for employees with high promotion focus.

## Materials and Methods

### Participants and Procedures

The data were collected from employees working at a large state-owned firm in Hubei Province in China. One of the authors contacted the senior director via a personal network and solicited his help to ask employees to participate in this study. With the help of this director, we visited the work sites and handed out the questionnaires during normal working hours. To minimize common method bias, we adopted a two-wave study approach to collect data (Podsakoff et al., 2012), with an interval of 2 weeks. At Time 1,300 employees responded to our survey, which included demographic information (e.g., gender, age, and education level) and the levels of leader inclusiveness and employees’ self-regulatory focus (e.g., promotion focus). After 2 weeks (at Time 2), these employees were invited to complete a second survey focused on thriving at work and taking charge. At Time 2, 247 questionnaires were collected. After excluding 31 incomplete questionnaires (e.g., more than half of the questions were not answered, the entire questionnaire had the same option, or the options showed regularity), the final sample included 206 employees, or a response rate of about 68.7%. Among the respondents, 49% were women and 51% were men. The average age was 29.2 years old. In terms of educational background, 39.4% had a Bachelor’s degree or higher.

### Measures

We used well-established scales to measure the constructs (e.g., leader inclusiveness, thriving at work, regulatory focus, and taking charge). Following the translation and back-translation procedure, we created a Chinese version of the scales for measuring these variables. All items in the study were rated on a 7-point Likert scale (1 = Strongly disagree to 7 = Strongly agree).

#### Leader Inclusiveness

To measure leader inclusiveness, we adopted the 9-item scale of Carmeli et al. (2010). The respondents were asked to report their perceptions of their leader’s openness, availability, and accessibility. Examples of items include “The manager is available for professional questions I want to ask him/her” and “The manager is attentive to new opportunities to improve the work process” (α = 0.93).

#### Thriving at Work

We used the 10-item scale developed by Porath et al. (2011). The scale included 10 items designed to reflect two dimensions. Examples of items include “I continue to learn more over time” and “I look forward to each new day” (α = 0.82).

#### Taking Charge

Parker and Collins’s (2010) 3-item scale for taking charge was used to measure this construct. Examples of items include “I try to improve procedures in my workplace” and “I try to introduce new work methods that are more effective” (α = 0.82).

#### Regulatory Focus

We measured promotion focus using the 9-item scale developed by Neubert et al. (2008). Examples of items include “I focus on completing my work tasks correctly to increase my job security” and “I frequently imagine how I will achieve my hopes and aspirations” (α = 0.90).

#### Control Variables

The gender, age, and education level of the employees were used as control variables in the analyses, each being related to thriving at work (Porath et al., 2011) and taking charge (Morrison and Phelps, 1999; Fuller et al., 2012). In addition, gender was treated as a dummy variable. Education level was divided into associate degree or below, Bachelor’s degree, Master’s degree, and doctoral degree.

### Analytic Strategy

We used SPSS 21.0 and Mplus 6.0 software (Muthén and Muthén, 2010) to test the hypotheses. In addition, the bootstrap method was used to estimate the confidence intervals (CIs) of the hypothesized mediation, moderation, and moderated mediation effects to determine their significance (Preacher et al., 2007, 2010).

### Measurement Models

Using Mplus 6.0 software, we conducted a series of confirmatory factor analyses (CFAs) to assess discriminant validity among the variables. Following the recommendations of Little et al. (2002), item parcels for leader inclusiveness, promotion focus, prevention focus, and thriving were created using an item-to-construct balance technique. No parcel was created for taking charge because of its small number of items (e.g., three items). The hypothesized four-item model (i.e., leader inclusiveness, thriving, taking charge, and promotion focus) showed an acceptable fit (χ^2^ = 155.84, df = 50; CFI = 0.93; TLI = 0.91; RMSEA = 0.09). The fit statistics for the hypothesized model showed a better fit than alternative measurement models. Specifically, the one-factor model (χ^2^ = 827.39, df = 90; CFI = 0.35; TLI = 0.25; RMSEA = 0.28) was not within the acceptable range. These CFA results suggested that the key constructs of this study had good discriminant validity.

## Results

### Descriptive Statistics

The means, standard deviations, and correlations of all of the variables are shown in Table 1. Leader inclusiveness was positively correlated with thriving at work (*r* = 0.31, *p* < 0.05) and taking charge (*r* = 0.25, *p* < 0.05). Furthermore, thriving at work was positively associated with taking charge (*r* = 0.44, *p* < 0.05). These results partly supported our hypothesized relationship.

**TABLE 1:** Means, standard deviations, and bivariate correlations among study variables.

**Variables**	**Mean**	**SD**	**1**	**2**	**3**	**4**	**5**	**6**	**7**
(1) Gender	1.51	0.50							
(2) Age	29.24	5.84	–0.08						
(3) Education level	2.18	0.95	–0.09	–0.10					
(4) Leader inclusiveness	4.89	0.96	–0.07	–0.06	0.04	(0.93)			
(5) Thriving at work	4.49	0.92	–0.02	–0.49	–0.05	0.31^∗∗^	(0.82)		
(6) Promotion focus	3.99	1.21	–0.18^∗∗^	0.00	–0.25^∗∗^	0.08	0.43^∗∗^	(0.90)	
(7) Taking charge	4.11	1.04	–0.05	–0.04	–0.14	0.25^∗∗^	0.44^∗∗^	0.29^∗∗^	(0.82)

**N* = 206. Internal consistency reliabilities are in parentheses on the diagonal. ^∗∗^*p* < 0.01 (two tailed).*

### Hypothesis Testing

To estimate the hypothesized model, we included gender, age, and education level with fixed effects in the mediating variable (thriving at work) and the dependent variable (taking charge). Tables 2, 3 report the results of our analyses.

**TABLE 2:** Analyses for thriving at work.

**Variables**	**Thriving at work**					
	**Model 1**		**Model 2**		**Model 3**	
	**Estimate**	**SE**	**Estimate**	**SE**	**Estimate**	**SE**
Gender	0.04	0.13	0.15	0.12	0.08	0.14
Age	0.004	0.01	–0.003	0.01	–0.00	0.01
Education level	–0.03	0.07	0.07	0.06	0.02	0.07
Leader inclusiveness	0.37^∗∗∗^	0.09	0.32^∗∗∗^	0.08	0.27^∗∗∗^	0.06
Promotion focus			0.29^∗∗∗^	0.08	0.27^∗∗^	0.06
Leader inclusiveness × promotion focus					0.09^∗^	0.05

*^∗^*p* < 0.05, ^∗∗^*p* < 0.01, ^∗∗∗^*p* < 0.001 (two tailed).*

**TABLE 3:** Analyses for taking charge.

**Variables**	**Taking charge**									
	**Model 4**		**Model 5**		**Model 6**		**Model 7**		**Model 8**	
	**Estimate**	**SE**	**Estimate**	**SE**	**Estimate**	**SE**	**Estimate**	**SE**	**Estimate**	**SE**
Gender	–0.11	0.16	–0.02	0.16	–0.11	0.15	–0.08	0.15	–0.08	0.14
Age	–0.01	0.01	–0.02	0.02	–0.01	0.01	–0.01	0.01	–0.00	0.01
Education level	–0.02	0.08	–0.17	0.08	–0.02	0.08	–0.12	0.08	–0.13	0.07
Leader inclusiveness	0.33^∗∗^	0.10			0.17	0.10	0.15	0.09	0.13	0.08
Thriving at work			0.47^∗∗^	0.17	0.41^∗^	0.16	0.39^∗∗^	0.13	0.45^∗^	0.10
Promotion focus							0.02	0.12	0.03	0.07
Leader inclusiveness × promotion focus									0.08^∗^	0.05

*^∗^*p* < 0.05, ^∗∗^*p* < 0.01 (two tailed).*

We first examined the direct effect of leader inclusiveness on taking charge. As Table 3 shows, leader inclusiveness was positively related to taking charge (*b* = 0.33, *p* < 0.01, Model 4, Table 3). Thus, H1 was supported. Then, we followed the guidelines of Preacher et al. (2010) and tested a path model specifying the indirect effect of leader inclusiveness on taking charge through thriving. Specifically, the results of the path model indicated that leader inclusiveness was positively related to taking charge (*b* = 0.17, *p* < 0.1, Model 6, Table 3), and thriving at work was related to taking charge (*b* = 0.41, *p* < 0.01, Model 6, Table 3). Furthermore, we used a parametric bootstrap procedure to estimate the CI around the indirect effect (Preacher et al., 2010). The results showed the positive indirect effect of leader inclusiveness on taking charge via thriving (estimate = 0.16, 95% CI [0.06, 0.28]). These results supported H2.

**FIGURE 2:**
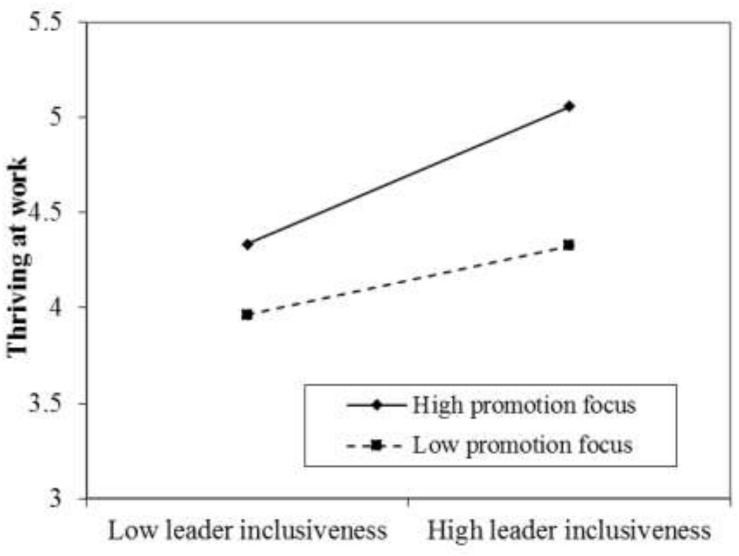
The moderating effect of promotion focus on leader inclusiveness for thriving at work.

For H3, we tested whether employees’ promotion focus can moderate the relationship between leader inclusiveness and thriving at work. We tested a model that included the direct effect of leader inclusiveness on thriving at work and the moderating effect of regular focus (i.e., promotion focus). The interaction term between inclusiveness and promotion focus was positively and significantly related to thriving at work (*b* = 0.09, *p* < 0.05, Model 3, Table 2). A parametric bootstrap procedure was used to estimate the CI around the indirect effect (Preacher et al., 2010) of promotion focus. The indirect effect was stronger when employees’ promotion focus was high (estimate = 0.37, 95% CI [0.25, 0.47]) and weaker when employees’ promotion focus was low (estimate = 0.18, 95% CI [0.01, 0.35]). Thus, H3 was supported. Following Aiken and West’s (1991) procedure, we plotted the interaction of leader inclusiveness and promotion focus at one standard deviation above and below the mean of promotion focus. As shown in Figure 2, leader inclusiveness was more positively correlated with thriving at work when promotion focus was high.

Finally, H4 is related to whether promotion focus can moderate the indirect effect of leader inclusiveness on taking charge via thriving at work. To predict the moderated mediation effect, we tested a model that included the mediating mechanism of thriving and a random slope between leader inclusiveness and thriving predicted by promotion focus. The interaction of leader inclusiveness and promotion focus was positively and significantly related to taking charge (*b* = 0.08, *p* < 0.05, Model 8, Table 3). A parametric bootstrap procedure was used to estimate the CI around the indirect effect (Preacher et al., 2007). As shown in Table 4, the indirect effect was stronger when employees’ promotion focus was high (estimate = 0.17, 95% CI [0.08, 0.30]) and weaker when employees’ promotion focus was low (estimate = 0.07, 95% CI [0.02, 0.16]). Thus, H4 was supported.

**TABLE 4:** Conditional indirect effect at specific values of promotion focus.

**Moderator**	**Level**	**Employee taking charge**		
		**Conditional indirect effect**	**SE**	**Bias-corrected 95% CI**
Promotion focus	Low	0.07^∗^	0.04	[0.02, 0.16]
	High	0.17^∗∗^	0.06	[0.08, 0.30]

*^∗^*p* < 0.05, ^∗∗^*p* < 0.01.*

## Discussion

This study developed a theoretical model explaining why and when leader inclusiveness influenced employee taking charge behavior. The results revealed that leader inclusiveness positively promoted employee taking charge behavior, while thriving at work functioned as a mediator in this relationship. Moreover, promotion focus moderated both the relationship between leader inclusiveness and thriving at work and the mediating effect of thriving at work in this relationship. When individuals’ promotion focus was high, the relationship and its mediating mechanism were stronger.

### Theoretical Implications

Our findings offer several theoretical implications. First, by adopting SDT as the theoretical foundation, this study built a conceptual model to test the effect of leader inclusiveness on taking charge. The results emphasized the key role of leader inclusiveness in fostering employee taking charge behavior, responding to the call for more research to explore the effect of leader inclusiveness on employee proactive behavior (Carmeli et al., 2010). In addition, by broadening the scope of employee outcomes encouraged by inclusive leaders, our findings provide a more complete picture of leader inclusiveness. Furthermore, leader inclusiveness has been an almost neglected antecedent of taking charge. Therefore, this study is one of the first to explore how leader inclusiveness enhances employee taking charge behavior. Moreover, our study contributes to research on employee proactive behavior. Leadership has been seen as playing a critical role in influencing employee proactive behavior. Previous research has shown that empowering leadership, self-sacrificial leadership, and leader support are positively related to proactive behavior (e.g., taking charge). This study, together with that of Javed et al. (2017), consistently emphasizes the significant role of this particular and effective leadership form – leader inclusiveness – in promoting employee proactive behavior.

Second, this study deepens our understanding of the relationship between leader inclusiveness and taking charge by examining thriving at work as a mediator. Exploring the mediating effect of thriving at work not only provides an important perspective to explain why employees under an inclusive leader are more likely to take charge, but also reveals the “black box” of the transmitting process from leader inclusiveness to employee taking charge behavior. In addition, by confirming the mediating role of thriving at work in the relationship between leader inclusiveness and taking charge, our study provides new evidence for the theoretical work of Spreitzer et al. (2005) that thriving is an important self-determination mechanism that transmits the effect of the unit context to individuals’ agentic behaviors. Furthermore, previous studies have examined certain psychological states as antecedents of taking charge, such as psychological empowerment (Kim et al., 2015), self-efficacy (Fuller et al., 2012), and organizational identification (Li R. et al., 2016). However, scholars have ignored thriving at work, which emphasizes individuals’ work-related state, including feeling energized and pursuing development and growth. Even within the broader research on employee proactive behavior, only limited studies have included thriving at work in their models. For example, Li M. et al. (2016) found that thriving at work transmitted the effect of empowering leadership to change-oriented organizational citizenship behavior. Nevertheless, they did not pay attention to the autonomous motivation implications of thriving at work. By adopting SDT, our study highlights the mediating effect of thriving at work in the relationship between leader inclusiveness and taking charge.

Third, this study reveals that promotion focus plays a particularly positive moderating role in the relationship between leader inclusiveness and thriving, and positively affects the mediating effect of thriving at work in the relationship between leader inclusiveness and taking charge. Specifically, we found that leader inclusiveness was strongly associated with thriving at work for employees with high promotion focus. Therefore, this study contributes to the literature on promotion focus by exploring its moderating effect on leader-follower exchange relationships, expanding previous research focused on its direct effect. The combination of inclusive leadership and promotion-focused employees creates a perfect fit, motivating employees to thrive at work and helping them take charge. In addition, this moderation is different from the moderating roles examined in previous research on taking charge, which did not consider leadership. Focusing on employees’ personal dispositions, previous research has investigated the moderating role of risk aversion (Li R. et al., 2016), autonomy orientation (Li M. et al., 2016), and anticipated costs (Burnett et al., 2013). These boundary conditions have rarely encompassed individuals’ regulatory focus. As a result, this study highlights the positive psychological state of individuals and deepens our understanding of the relationship between leader inclusiveness and taking charge.

### Practical Implications

These findings have managerial implications for cultivating taking charge among employees. First, we showed that leader inclusiveness plays a vital role in motivating employees to take charge. Thus, to enhance this desirable proactive behavior among employees, organizations should set up effective procedures for selecting and recruiting inclusive managers and should provide supervisors with training and development programs to help them learn to initiate inclusive management and encourage their employees. These human resource practices, which educate leaders to understand the effectiveness of inclusiveness and encourage them to behave more inclusively, are essential for employees to experience psychological growth and development and to take charge. In the Asian cultural context, which has rigid hierarchies and high power distance between leaders and subordinates, an inclusive leader is needed to ignite employees’ enthusiasm and encourage them to adopt proactive behavior. Second, the results indicated that thriving at work, which is a mental state of “vitality” and “learning,” precedes taking charge behavior. Because of its important mediating role in promoting employee taking charge behavior, managers should provide a suitable climate and conditions to maximize thriving at work. In addition, managers should monitor their employees’ psychological state, which is a useful way to check whether inclusive management can promote employee taking charge behavior.

Finally, the moderating effect of promotion focus on the relationship between leader inclusiveness and thriving at work suggests that managers should pay attention to their employees’ personality and should better understand them. For employees with high promotion focus, leaders will be effective in motivating them to thrive by being inclusive. Conversely, for employees with low promotion focus, the effect will be less significant.

### Limitations and Future Research

This study has several limitations. First, although the two-wave questionnaire survey with a 2-week interval recommended by Podsakoff et al. (2012) was adopted to avoid common method bias, it was not truly longitudinal and could lead to reverse causation. A more longitudinal method with multiple time points should be used to investigate the possible dynamic relationships between these variables. Second, our study examined the effect of leader inclusiveness on employee taking charge behavior at the individual level. However, evidence has shown that leadership can be aggregated at the team level and that variables at different levels can influence each other. Future research should use multilevel and cross-level designs to test the effect of leader inclusiveness on taking charge. Third, generalization of the results was difficult, as the data were collected from only one company in China. Investigations with data from multiple contexts are needed to test the influence of culture on our proposed hypotheses. Finally, although we tested important contextual and individual factors related to taking charge, there are probably other moderators and mediators that can explain the effect on taking charge. For example, future research should pay attention to psychological safety (Carmeli et al., 2010) and leader-member exchanges as mediators in the relationship between leader inclusiveness and taking charge. In addition, we suggest that autonomy (Li M. et al., 2016), risk aversion (Li R. et al., 2016), and participation costs (Burnett et al., 2013), which are associated with taking charge, should be examined as moderators.

## Ethics Statement

This study was carried out in accordance with the recommendations of the Ethic Committee of the School of Business Administration, Zhongnan University of Economics and Law, with written informed consent from all subjects. All subjects gave written informed consent in accordance with the Declaration of Helsinki. The protocol was approved by the Ethic Committee of the School of Business Administration, Zhongnan University of Economics and Law.

## Author Contributions

HW proposed the research idea, designed the study, and collected the data. NL and Q-YG drafted the manuscript and revised the article critically.

## Conflict of Interest

The authors declare that the research was conducted in the absence of any commercial or financial relationships that could be construed as a potential conflict of interest.
